# Altered Local Brain Amplitude of Fluctuations in Patients With Myotonic Dystrophy Type 1

**DOI:** 10.3389/fnagi.2021.790632

**Published:** 2021-12-10

**Authors:** Pei Huang, Xing-Hua Luan, Zhou Xie, Meng-Ting Li, Sheng-Di Chen, Jun Liu, Xi-Ze Jia, Li Cao, Hai-Yan Zhou

**Affiliations:** ^1^Department of Neurology and Institute of Neurology, Ruijin Hospital, Shanghai Jiao Tong University School of Medicine, Shanghai, China; ^2^Department of Neurology, Shanghai Jiao Tong University Affiliated Sixth People’s Hospital, Shanghai, China; ^3^School of Information and Electronics Technology, Jiamusi University, Jiamusi, China; ^4^Key Laboratory of Intelligent Education Technology and Application of Zhejiang Province, Zhejiang Normal University, Jinhua, China

**Keywords:** amplitude of low-frequency fluctuations, resting-state functional MRI, myotonic dystrophy type 1, local brain function, spontaneous brain activity

## Abstract

This study is aimed at investigating the characteristics of the spontaneous brain activity in patients with myotonic dystrophy type 1 (DM1). A total of 18 patients with DM1 and 18 healthy controls (HCs) were examined by resting-state functional MRI. Combined methods include amplitude of low-frequency fluctuations (ALFFs), the fractional amplitude of low-frequency fluctuations (fALFFs), and Wavelet transform-based ALFFs (Wavelet-ALFFs) with standardization, percent amplitude of fluctuation (PerAF) with/without standardization were applied to evaluate the spontaneous brain activity of patients with DM1. Compared with HCs, patients with DM1 showed decreased ALFFs and Wavelet-ALFFs in the bilateral precuneus (PCUN), angular gyrus (ANG), inferior parietal, but supramarginal and angular gyri (IPL), posterior cingulate gyrus (PCG), superior frontal gyrus, medial (SFGmed), middle occipital gyrus (MOG), which were mainly distributed in the brain regions of default mode network (DMN). Decreased ALFFs and Wavelet-ALFFs were also seen in bilateral middle frontal gyrus (MFG), inferior frontal gyrus, opercular part (IFGoperc), which were the main components of the executive control network (ECN). Patients with DM1 also showed decreased fALFFs in SFGmed.R, the right anterior cingulate and paracingulate gyri (ACGR), bilateral MFG. Reduced PerAF in bilateral PCUN, ANG, PCG, MOG, and IPLL as well as decreased PerAF without standardization in PCUNR and bilateral PCG also existed in patients with DM1. In conclusion, patients with DM1 had decreased activity in DMN and ECN with increased fluctuations in the temporal cortex and cerebellum. Decreased brain activity in DMN was the most repeatable and reliable with PCUN and PCG being the most specific imaging biomarker of brain dysfunction in patients with DM1.

## Introduction

Myotonic dystrophy type 1 (DM1) is an autosomal dominant hereditary disease caused by the expansion of the cytosine thymine guanine triplet repeats within the myotonic dystrophy protein of the kinase (DMPK) gene. The prevalence of DM1 ranges from 0.5 to 18.1 per 100,000 population, making it the most common muscular dystrophy disease (Theadom et al., 2014). DM1 can present at any age but is typically diagnosed in adults with many different ways of presentation, such as facial and distal muscle weakness, along with grip myotonia. It was primarily recognized as a disease-affecting muscular function, whereas many patients also experienced multiple symptoms including cognitive impairment, apathy, sleep disorders, and behavioral disturbances (Theadom et al., 2014; Okkersen et al., 2017a).

Neurological involvement in the DM1 has aroused lots of interest in the recent years and several brain MRI studies have reported white matter lesions and brain atrophy (Minnerop et al., 2011; Baldanzi et al., 2016; Zanigni et al., 2016; Okkersen et al., 2017b; Koscik et al., 2021; Leddy et al., 2021; Lopez-Titla et al., 2021). Diffusion tensor imaging studies have demonstrated widespread white matter lesions with reduced fractional anisotropy (FA) and increased mean diffusivity in patients with DM1 compared with controls (Cabada et al., 2017; Yoo et al., 2017; van Dorst et al., 2019). Widespread reduction of gray matter volume (including frontal, temporal, parietal, and occipital cortices, as well as deep gray matter structures and cerebellum) was found in patients with DM1 using the voxel-based morphometry method (Minnerop et al., 2011; Baldanzi et al., 2016; Zanigni et al., 2016; Hamilton et al., 2018; Park et al., 2018; Gliem et al., 2019; Labayru et al., 2019, 2020; Serra et al., 2020a; Cabada et al., 2021). Recent studies have investigated abnormalities in gray matter structural networks using graph theoretical analysis. Frontal disconnection and increased parietal-cerebellar connectivity were identified in the brains of the patients (Serra et al., 2016a,b; Cabada et al., 2017; Sugiyama et al., 2017; Yoo et al., 2017). However, even with such widespread white matter lesions and brain atrophy, some general cognitive function of patients with DM1 were still preserved (Gaul et al., 2006). It indicates that structural measurements are not able to accurately reflect the changes of the brain function, investigation of brain functional changes in patients with DM1 is urgent.

Till now, very few studies have focused on the functional analysis of brain MRI in DM1 patients (Serra et al., 2014, 2020b). Previous studies using motor-task-related fMRI had found altered activation patterns in bilateral motor regions in patients with DM1 (Caramia et al., 2010; Toth et al., 2015). The resting-state fMRI study focusing on brain functional connectivity had reported abnormal functional connectivity in default mode network (DMN) in relation to deficits of personality traits and social cognition (Serra et al., 2014). Although brain functional connectivity analysis gave us an in-depth understanding of brain impairments in patients with DM1, it could not reflect local brain function change, which made it difficult to localize the responsible brain damage.

To specifically localize the brain regions with abnormal activity, the amplitude of low-frequency fluctuations (ALFFs) is a reliable approach to monitor spontaneous neuronal fluctuations and can reflect cerebral physiological states and local brain functions (Zang et al., 2007). However, each method has its own advantages and limitations. As we know, ALFFs have been widely applied to the resting-state fMRI studies of the neurological diseases, but it could be easily influenced by noise signals (Zuo et al., 2010). To effectively inhibit non-specific signal components of resting-state fMRI, fractional amplitude low-frequency fluctuations (fALFFs) are applied to measure a range of low frequency (0.01–0.08 Hz) divided by the entire frequency range (Zou et al., 2008). Compared with ALFFs calculated with fast Fourier transform (FFT), wavelet transform (WT)-based ALFFs (Wavelet-ALFFs) is more effective in depicting the complex time series (Luo et al., 2020). In addition, the percent amplitude of fluctuation (PerAF) was proved to have good reliability in investigating the abnormal BOLD signal in resting-state fMRI (Jia et al., 2020).

Thus, this study aims to investigate the local spontaneous brain activity of patients with DM1 using combined methods of the amplitude of low-frequency fluctuations, such as ALFFs, fALFFs, Wavelet-ALFFs, PerAF with/without global mean standardization. We want to discover the most reliable abnormal changes of the local brain function in patients with DM1.

## Materials and Methods

### Participants

Diagnosis of DM1 was based on clinical features and electromyographic evidence of myopathy and myotonia. A total of 18 patients with DM1 were enrolled and all the cases were genetically confirmed. The inclusion criteria for patients were as follows: (1) right-handed according to Edinburgh Handedness Inventory (Oldfield, 1971); (2) age ranging from 20 to 80 years; and (3) genetically confirmed cases of DM1. Exclusion criteria included: (1) history of drug and alcohol abuse; (2) brain damage, such as head trauma and history of stroke; (3) other diseases that cause muscle weakness, such as myositis, myasthenia gravis, and peripheral neuropathy; and (4) MRI incompatibility. A total of 18 healthy controls (HCs) demographically matched with patients in terms of age, sex, and years of education were recruited.

All the participants were fully informed and signed written consent forms. This study was approved by the Ethic Committee of the Ruijin Hospital Affiliated to the Shanghai Jiao Tong University School of Medicine, and registered on the Chinese clinical trial registry (ChiCTR2000032978).

### Clinical Examinations

All the participants were evaluated by neurologists. Cognition and depression were assessed by the Mini-Mental State Examination (MMSE) and the Hamilton Depression Scale-17 (HAMD-17). The patients also accepted electromyographic testing and genetic testing.

### Magnetic Resonance Imaging Data Acquisition

A total of 18 patients with DM1 and 18 HC participated in rs-fMRI scan on a 3.0 Tesla GE Medical System (GE Healthcare, Little Chalfont, United Kingdom) scanner. During the scan, the subjects were asked to remain motionless and awake with their eyes closed. For each participant, 210 functional images were collected using blood oxygen level dependent (BOLD) sequence [repetition time (TR) = 2,000 ms; echo time (TE) = 30 ms; flip angle = 90°; 35 slices; matrix = 64 × 64; voxel size = 3.75 mm^3^ × 3.75 mm^3^ × 4 mm^3^]. Then, the high-resolution, three-dimensional, T1-weighted structural images (TR = 5.5 ms; TE = 1.7 ms; flip angle = 12°; matrix = 256 × 256; voxel size = 1 mm^3^ × 1 mm^3^ × 1 mm^3^) were acquired for the registration and normalization of the functional images.

### Magnetic Resonance Imaging Data Pre-processing

Image data pre-processing was carried out using RESTplus V1.24^1^ (Jia et al., 2019) and SPM12.^2^ The pre-processing steps included: (1) removing the first 10 time points; (2) slice timing correction; and (3) realigning. Participants of head motion exceeding 3 mm or 3° were excluded. Finally, two patients with DM1 were excluded; (4) First, an individual structural image was coregistered to the mean functional image, and then it was segmented into tissue segmentation of the structural images. The Diffeomorphic Anatomical Registration Through Exponentiated Lie Algebra (DARTEL) tool was used to compute the transformation from individual space to MNI space and vice-versa (resampling voxel size = 3 mm × 3 mm × 3 mm); (5) Smooth. Spatial smoothing with a Gaussian kernel of 6 mm full-width at half-maximum (FWHM) to explore the influence of different smooth kernel on the results, we also smoothed the fMRI data with a 4 mm FWHM; (6) Removing the linear trend of the time series; (7) Regressing out nuisance variables, including Friston-24 head motion parameters (Friston et al., 1996; Jenkinson et al., 2002), the cerebrospinal flow signals, and white matter signals. The mean value of the time series of each voxel was added back in this step; considering the influence of the global brain signals (Macey et al., 2004; Fox et al., 2009), we also regressed out the global mean signals to see the data stability; (8) Band-pass filtering (0.01–0.08 Hz), this step of pre-processing was only done in PerAF.

### Magnetic Resonance Imaging Data Processing

Magnetic resonance imaging data of 16 patients with DM1 and 18 HC were enrolled into the next analysis. The ALFFs and fALFFs analyses were performed using the RESTplus V1.24. After pre-processing, the time series of each voxel was transformed to the frequency domain by the fast Fourier transform (FFT), and the power spectrum was obtained. The square root was calculated at each frequency of the power spectrum. The average square root across 0.01–0.08 Hz was taken as ALFFs of each voxel (Zang et al., 2007). The ratio of the sum of amplitude within the 0.01–0.08 Hz to that of the whole frequency band was calculated as fALFFs (Zou et al., 2008). For standardization, the ALFFs and fALFFs value of each voxel was divided by the global mean ALFFs and the global mean fALFFs within the brain mask separately.

The PerAF of each voxel was calculated as follows (Jia et al., 2020),

$$P\,e\,r\,A\,F=\frac{1}{n}\,\sum_{i=1}^{n}\left|\frac{X_{i}-\mu }{\mu }\right|\times 100\%$$

$$\mu =\frac{1}{n}\,\sum_{i=1}^{n}X_{i}$$

where *X*_*i*_ is the signal intensity of the *i*_*th*_ time point, *n* is the total number of time points of the time series, and μ is the mean value of the time series. The PerAF of each voxel was then divided by the global mean of the PerAF values for standardization.

Wavelet-ALFFs calculation was based on continuous WT (CWT). Wavelet-ALFFs was calculated by first adding up the wavelet coefficients at all time points for each frequency point, and the averaged coefficient across a given frequency band was then obtained as defined later (Luo et al., 2020):

$$W\,a\,v\,e\,l\,e\,t-A\,L\,F\,F=\frac{1}{m}\,\sum_{i=1}^{n}\left|C\,W\,T_{i,j}\right|,j=s_{1}\,\ldots \,s_{m}$$

where |*CWT*_*i*,*j*_| denotes the absolute value of wavelet coefficient at time point *i* at a given frequency point *j*;*n* denotes the total amount of wavelet coefficient at a given frequency point; and *m* denotes the total number of frequency points across a given frequency band. The Wavelet-ALFFs of each voxel was then divided by the global mean of the Wavelet-ALFFs values for standardization. In this study, we calculated the Wavelet-ALFFs in the conventional frequency band of 0.01–0.08 Hz.

### Statistical Analysis

The two-sample *t*-test was performed in the demographic data of both the groups in SPSS (version 24.0, Armonk, New York, United States). All the tests of demographics were two-tailed and *p* < 0.05 was considered significant. The ALFFs, fALFFs, and Wavelet-ALFFs with standardization, PerAF with/without standardization were analyzed by an independent two-sample *t*-test in the DPABI V5.1^3^ (Yan et al., 2016). The permutation test with a threshold-free cluster enhancement (TFCE) (Chen et al., 2018) (number of permutations = 5,000) method was used in the two-sample *t*-test, and the comparison was done between patients and control group in gray matter mask. A *P* < 0.01 and cluster size threshold ≥10 voxels was considered statistically significant (FWE corrected). In addition, since the head motion is an important confounding factor in fMRI studies (Satterthwaite et al., 2012; Zeng et al., 2014), the frame-wise displacement (FD) parameter was regressed out in the two-sample *t*-test of all the five metrics, and results were thresholded with the TFCE (*P*_FWE_ < 0.01, number of permutations was set as 5,000 with a minimum cluster size = 10).

## Results

### Demographics and Clinical Characteristics

A total of 18 patients with DM1 and 18 HCs were enrolled in the study, while 2 patients with DM1 were excluded because of head motion. 16 patients with DM1 and 18 HCs were included into the final analysis. They were matched in age (*p* = 0.114), gender (*p* = 0.510), and education (*p* = 0.121), but patients with DM1 showed significantly lower score in MMSE (*p* < 0.001) and higher score in HAMD-17 (*p* < 0.001) than HCs (Table 1).

**TABLE 1:** Demographics and clinical characteristics of the subjects.

Characteristics	DM1 (*n* = 16)	HC (*n* = 18)	*p* value
Age (y)	48.44 ± 14.14	41.50 ± 10.67	0.114
Gender (M/F)	10/6	9/9	0.510
Education (y)	13.00 ± 3.16	14.56 ± 2.53	0.121
Disease duration (y)	6.19 ± 4.82	—	—
MMSE	27.06 ± 1.84	29.17 ± 0.92	<0.001
HAMD-17	6.00 ± 4.23	2.06 ± 0.80	<0.001

*Abbreviations: DM1, myotonic dystrophy type 1; HC, healthy control; MMSE, Mini-Mental State Examination; HAMD-17, Hamilton Depression Scale-17.*

### Group Differences in Amplitude of Low-Frequency Fluctuations

**FIGURE 1:**
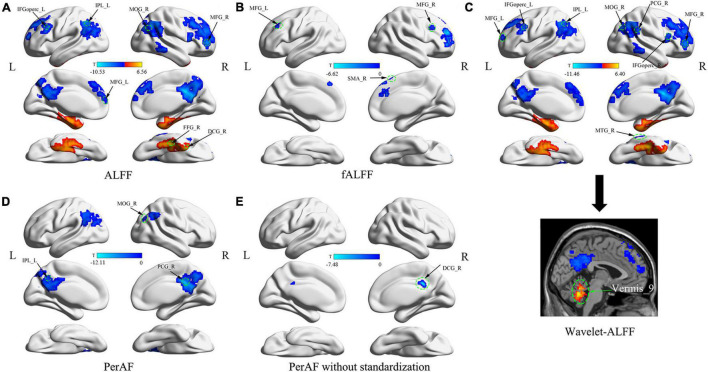
Abnormal brain fluctuations of patients with myotonic dystrophy type 1 (DM1) compared with healthy controls. **(A)** Differences in ALFFs; **(B)** Differences in fALFF; **(C)** Differences in Wavelet-ALFF; **(D)** Differences in PerAF; **(E)** Differences in PerAF without standardization. Abbreviations: ALFFs, amplitude of low-frequency fluctuations; fALFFs, fractional amplitude of low-frequency fluctuations; Wavelet-ALFF, wavelet transform-based ALFF; PerAF, percent amplitude of fluctuation.

Compared with HC, patients with DM1 showed increased ALFFs in the left inferior temporal gyrus (ITGL), left parahippocampal gyrus (PHGL), right fusiform gyrus (FFGR), right inferior cerebellum (Cerebelum_8R); and decreased ALFFs in the right precuneus (PCUNR), bilateral angular gyrus (ANG), left inferior parietal, but supramarginal and angular gyri (IPLL), left posterior cingulate gyrus (PCGL), bilateral superior frontal gyrus, medial (SFGmed), right middle occipital gyrus (MOGR). Decreased ALFFs were also seen in bilateral middle frontal gyrus (MFG), left inferior frontal gyrus, opercular part (IFGopercL) and also the right median cingulate and paracingulate gyri (DCGR) (Table 2 and Figure 1A).

**TABLE 2:** Brain regions showing differences in ALFFs, fALFFs, Wavelet-ALFFs, and PerAF with/without standardization between the groups.

Metric	Cluster	Brain regions	L/R	Cluster size	BA	Peak MNI coordinates			Peak intensity (*t*)
						X	Y	Z	
ALFF	1	FFG	R	2200	/	42	–24	–24	6.5566
		Cerebelum_8	R	232					
		ITG	L	160					
		PHG	L	71					
	2	MOG	R	689	/	36	–72	36	–7.4354
		ANG	R	229					
		PCUN	R	82					
	3	DCG	R	903	31	3	–42	39	–10.5306
		PCUN	R	305					
		PCG	L	75					
	4	MFG	R	1309	/	42	48	3	–6.7737
		SFGmed	L	149					
		SFGmed	R	141					
	5	IPL	L	580	/	–45	–54	45	–6.7280
		ANG	L	176					
	6	MFG	L	22	10	–27	60	15	–5.7711
	7	IFGoperc	L	73	/	–42	18	33	–4.7665
		MFG	L	34					
fALFF	1	SMA	R	380	/	6	24	63	–6.6199
		MFG	R	155					
		SFGmed	R	63					
		ACG	R	5					
	2	MFG	R	10	9	39	21	33	–5.2016
	3	MFG	L	20	9	–45	27	39	–4.6801
Wavelet-ALFF	1	Vermis_9	/	2102	/	–3	–57	–33	6.3959
		Cerebelum_8	L	233					
		ITG	L	148					
		PHG	L	67					
	2	MTG	R	65	/	66	–42	0	–5.4868
	3	PCG	R	752	/	6	–42	30	–11.4574
		PCUN	R	241					
		PCUN	L	183					
	4	MFG	R	1113	/	42	48	3	–6.7995
		SFGmed	L	170					
		SFGmed	R	134					
		ACG	R	7					
	5	MFG	L	11	10	–27	60	15	–5.6510
	6	IPL	L	522	/	–45	–54	45	–7.0722
		ANG	L	164					
		MOG	L	143					
	7	IFGoperc	R	77	/	48	15	15	–5.2355
	8	MOG	R	494	/	36	–72	36	–7.5466
		ANG	R	195					
		IPL	R	113					
	9	IFGoperc	L	63	/	–42	18	33	–4.7363
PerAF	1	PCG	R	773	31	3	–42	30	–12.113
		PCUN	R	244					
		PCUN	L	186					
		PCG	L	72					
	2	IPL	L	484	40	–45	–51	42	–6.6327
		MOG	L	170					
		ANG	L	149					
	3	MOG	R	208	/	36	–72	36	–6.5362
		ANG	R	90					
PerAF without standardization	1	DCG	R	38	31	3	–39	33	–7.4807
		PCG	R	6					
		PCG	L	3					
		PCUN	R	1					

*Abbreviations: MNI, Montreal Neurological Institute, BA, Brodmann area; ALFF, amplitude of low-frequency fluctuations; fALFF, fractional amplitude of low-frequency fluctuations; Wavelet-ALFF, wavelet transform-based ALFF; PerAF, percent amplitude of fluctuation; FFG, fusiform gyrus; ITG, inferior temporal gyrus; PHG, parahippocampal gyrus; MOG, middle occipital gyrus; ANG, angular gyrus; PCUN, precuneus; DCG, median cingulate and paracingulate gyri; PCG, posterior cingulate gyrus; MFG, middle frontal gyrus; SFGmed, superior frontal gyrus medial; IPL, inferior parietal; IFGoperc, inferior frontal gyrus, opercular part; SMA, supplementary motor area; ACG, anterior cingulate and paracingulate gyri; MTG, middle temporal gyrus.*

### Group Differences in Fractional Amplitude of Low-Frequency Fluctuations

Compared with HC, patients with DM1 showed decreased fALFFs in SFGmedR. The right anterior cingulate and paracingulate gyri (ACG.R), bilateral MFG also presented decreased fALFFs as well as the right supplementary motor area (SMA.R) (Table 2 and Figure 1B).

### Group Differences in Wavelet-Amplitude of Low-Frequency Fluctuations

Compared with HC, patients with DM1 showed increased Wavelet-ALFFs in ITGL, PHGL, Cerebelum_8L, and Vermis_9 and decreased Wavelet-ALFFs in bilateral PCUN, bilateral ANG, bilateral IPL, PCGR, bilateral SFGmed, and bilateral MOG. Bilateral MFG, bilateral IFGoperc, ACGR, and the right middle temporal gyrus (MTGR) also presented decreased Wavelet-ALFFs (Table 2 and Figure 1C).

### Group Differences in Percent Amplitude of Fluctuation

Compared with HC, patients with DM1 showed decreased PerAF in bilateral PCUN, bilateral ANG, IPLL, bilateral PCG, and bilateral MOG (Table 2 and Figure 1D).

### Group Differences in Percent Amplitude of Fluctuation Without Standardization

Compared with HC, patients with DM1 showed decreased PerAF without standardization in PCUN.R and bilateral PCG. In addition, DCG.R also presented decreased PerAF without standardization (Table 2 and Figure 1E).

### Data Stability

The result patterns of all the 5 metrics before and after the regression of FD parameters were similar, which indicated weak influence of head motion on the results (see Supplementary Figures 1, 4, 7, 10, 13). After regressing out the global mean signals, the result patterns of all the five metrics also remained similar (see Supplementary Figures 2, 5, 8, 11, 14). The result patterns of all the five metrics with two FWHM Gaussian kernel were consistent, which also indicated the stability of our results (see Supplementary Figures 3, 6, 9, 12, 15).

## Discussion

This study found that patients with DM1 had decreased amplitude of brain spontaneous activity in PCUN, ANG, IPL, PCG, SFGmed, and MOG, which were the major components of DMN. Spontaneous brain fluctuations in MFG, IFGoperc, ACG, and MTG, which were the main components of the executive control network (ECN), also showed a decreased pattern. Other brain regions, such as SMA and DCG also presented decreased brain activity in patients with DM1 compared with HC. Abnormal increased brain fluctuations were found in ITG, PHG, FFG, cerebelum_8, and vermis_9. Among all the abnormal changes in amplitude of low-frequency fluctuations based on four different methods, such as ALFFs, fALFFs, Wavelet-ALFFs and PerAF with/without standardization, decreased brain activity in DMN was the most reliable and robust result.

Default mode network includes bilateral ventromedial prefrontal cortex, dorsomedial prefrontal cortex, posterior cingulate cortex and adjacent precuneus, as well as the lateral parietal cortex (Biswal et al., 1995; Brown et al., 2019). The DMN is a set of regions that are most active at rest and decrease in activity during externally directed tasks (Brown et al., 2019). The DMN is thought to be primarily responsible for internally focused thought processes, such as autobiographical memory and experience of the self, which is an important contributor to executive function performance (Chand et al., 2017). Age-related reduction in DMN deactivation is associated with poorer executive function task performance (Brown et al., 2015). Patients with DM1 were reported to have significant decrease in executive function (Gaul et al., 2006; Filli et al., 2020). Our study found decreased brain activity in PCUN, ANG, IPL, PCG, SFGmed, and MOG, which mainly distributed in brain regions of the DMN. Based on the results from four different methods of analyzing amplitude of low-frequency fluctuations, decreased brain activity in DMN was the most reliable and robust. Previous studies of brain functional connectivity using rs-fMRI reported abnormal functional connectivity in DMN, which was related to patients with DM1 deficits of personality traits and social cognition (Buckner et al., 2005; Lenzoni et al., 2020). This study further localized the abnormally functioned brain regions by analyzing the local brain fluctuations, which helped us further understanding the pathogenesis of the disease.

Brain regions correlated with ECN, such as MFG, IFGoperc, ACG, and MTG, also presented decreased brain fluctuations. Regions within the ECN include anterior cingulate cortex/pre-supplementary motor area (ACC/pre-SMA), dorsolateral prefrontal cortex, inferior frontal junction, anterior insular cortex, dorsal premotor cortex, and posterior parietal cortex (Cole and Schneider, 2007; Xu et al., 2020). ECN is widely reported to be more activated for externally directed higher-order cognitive functions, such as attention, working memory, and decision-making (Bressler and Menon, 2010). The functional cross-talk between DMN and ECN networks mirrors the integrity of cognitive processing and is directly related to brain development (Fu et al., 2021; Xue et al., 2021). Low DMN deactivation and high ECN activation were suggested to be the main predictors of executive dysfunction (Della Rosa et al., 2021). This study indicated that ECN, especially such brain regions as MFG, IFGoperc, ACG, and MTG might play an important role in patients with DM1 brain dysfunction.

Other brain regions such as SMA and DCG also presented decreased the brain activity in patients with DM1 compared with HC. Considered as a main component of cognitive control network, SMA might connect with ECN through pre-SMA (Niendam et al., 2012), participate in automatic motor actions (Nee et al., 2007), and modulate interhemispheric interactions (Welniarz et al., 2019). Abnormal functional connectivity between DCG and dorsal anterior insular was related to deficits in attention/working memory and visuospatial function (Pan et al., 2021). Further investigation is needed to explore the relationship between abnormal brain activity in SMA and DCG and the cognitive deficits of patients with DM1.

Abnormal pattern of the brain activity in patients with DM1 also includes increased brain fluctuations in ITG, PHG, FFG, cerebelum_8, and vermis_9, which are mainly distributed in the temporal cortex and cerebellum. Previous studies demonstrate that ITG, PHG, and FFG were associated with emotion processing (Liao et al., 2011; Müller et al., 2012). The lobule VIII of the cerebellum was connected with the sensorimotor network and the vermis was involved in emotion function (Schmahmann, 2019). Although their underlying pathophysiological mechanism is unclear, abnormal local brain fluctuations in ITG, PHG, FFG, cerebelum_8, and vermis may correlate with emotional deficits of patients with DM1.

Aforementioned results were confirmed with the combination of different methods of evaluating local brain fluctuation. As we know, each method has its advantages and limitations. In our study, decreased brain activity in DMN and ECN, especially in PCUN and PCG, was consistent among different methods. What is more that the results were stable after considering the influence of head motion, global brain signals, and different smooth kernel. Thus, decreased local brain fluctuation in PCUN and PCG was considered the most reliable and robust change in patients with DM1.

## Limitations

The limitation of this study is the relatively small sample size, and larger cohort is needed to confirm the results in the future studies. However, the prevalence of DM1 ranges from 0.5 to 18.1 per 100,000 population and belongs to rare disease, and the local brain function of patients with DM1 has not been investigated previously. What is important is that reliable and stable changes in PCUN and PCG of patients with DM1 are confirmed based on the consistent results among different methods. This study provides good evidence of altered amplitude of fluctuations in DMN and ECN, especially in PCUN and PCG areas. We attend to further explore the cognitive and emotional changes in patients with DM1, and decipher the relationship between clinical presentations and brain functional changes in a larger scale sample.

## Conclusion

Patients with DM1 had decreased activity in DMN and ECN with increased fluctuations in the temporal cortex and cerebellum. Decreased brain activity in DMN was the most reliable with PCUN and PCG being the most robust brain regions.

## Data Availability Statement

The raw data supporting the conclusions of this article will be made available by the authors, without undue reservation.

## Ethics Statement

The studies involving human participants were reviewed and approved by the Ethics Committee of Ruijin Hospital Affiliated to Shanghai Jiao Tong University School of Medicine. The patients/participants provided their written informed consent to participate in this study.

## Author Contributions

H-YZ, LC, PH, X-HL, and X-ZJ gave study conceptualization and design. PH and X-HL were involved in data collection. PH, ZX, X-HL, X-ZJ, and M-TL helped with data analysis and interpretation. LC, H-YZ, S-DC, and JL contributed to the supervision of the study procedures. PH and ZX contributed to drafting the manuscript. All authors contributed to the final version of the manuscript.

## Conflict of Interest

The authors declare that the research was conducted in the absence of any commercial or financial relationships that could be construed as a potential conflict of interest. The editor G-YY has declared a shared parent affiliation with the authors PH, X-HL, S-DC, JL, LC, and H-YZ at the time of review.

## Publisher’s Note

All claims expressed in this article are solely those of the authors and do not necessarily represent those of their affiliated organizations, or those of the publisher, the editors and the reviewers. Any product that may be evaluated in this article, or claim that may be made by its manufacturer, is not guaranteed or endorsed by the publisher.

## Footnotes

1
http://restfmri.net/forum/restplus

2
https://www.fil.ion.ucl.ac.uk/spm/

3
http://rfmri.org/dpabi
