# Supplementary material for: Altered Local Brain Amplitude of Fluctuations in Patients With Myotonic Dystrophy Type 1
Source: Front Aging Neurosci. 2021 Dec 10;13:790632. doi: 10.3389/fnagi.2021.790632 (PMC8703136; doi:10.3389/fnagi.2021.790632)
Supplement: Supplementary file 1 [file Data_Sheet_1.DOCX]

**Supplementary materials**

**Figure S1.** The ALFF differences between two groups (Frame-wise displacement (FD) parameters were regressed out in the statistical analysis).

**Figure S2.** The ALFF differences between two groups (Smooth kernel was 4mm).

**Figure S3.** The ALFF differences between two groups (The global mean signals were regressed out in the preprocess).

**Figure S4.** The fALFF differences between two groups (Frame-wise displacement (FD) parameters were regressed out in the statistical analysis).

**Figure S5.** The fALFF differences between two groups (Smooth kernel was 4mm).

**Figure S6.** The fALFF differences between two groups (The global mean signals were regressed out in the preprocess).

**Figure S7.** The PerAF differences between two groups (Frame-wise displacement (FD) parameters were regressed out in the statistical analysis).

**Figure S8.** The PerAF differences between two groups (Smooth kernel was 4mm).

**Figure S9.** The PerAF differences between two groups (The global mean signals were regressed out in the preprocess).

**Figure S10.** The PerAF (without standardization) differences between two groups (Frame-wise displacement (FD) parameters were regressed out in the statistical analysis).

**Figure S11.** The PerAF (without standardization) differences between two groups (Smooth kernel was 4mm).

**Figure S12.** The PerAF (without standardization) differences between two groups (The global mean signals were regressed out in the preprocess).

**Figure S13.** The Wavelet-ALFF differences between two groups (Frame-wise displacement (FD) parameters were regressed out in the statistical analysis).

**Figure S14.** The Wavelet-ALFF differences between two groups (Smooth kernel was 4mm).

**Figure S15.** The Wavelet-ALFF differences between two groups (The global mean signals were regressed out in the preprocess.


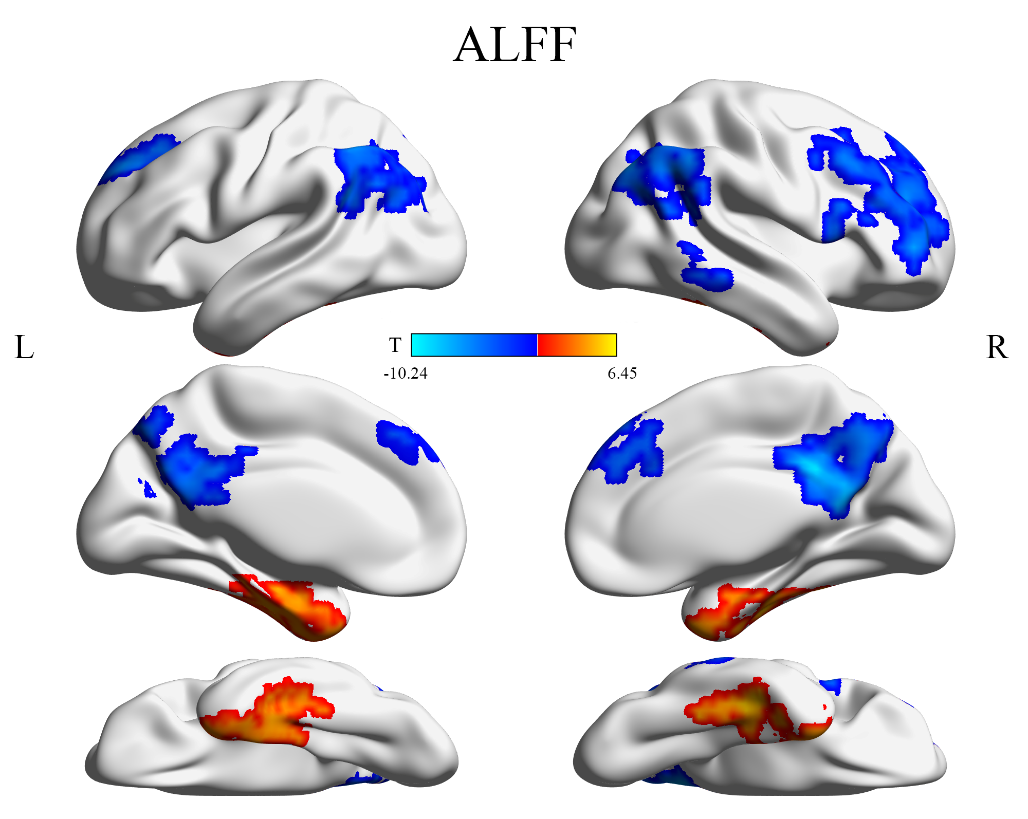


**Figure S1.** The ALFF differences between two groups (Frame-wise displacement (FD) parameters were regressed out in the statistical analysis).


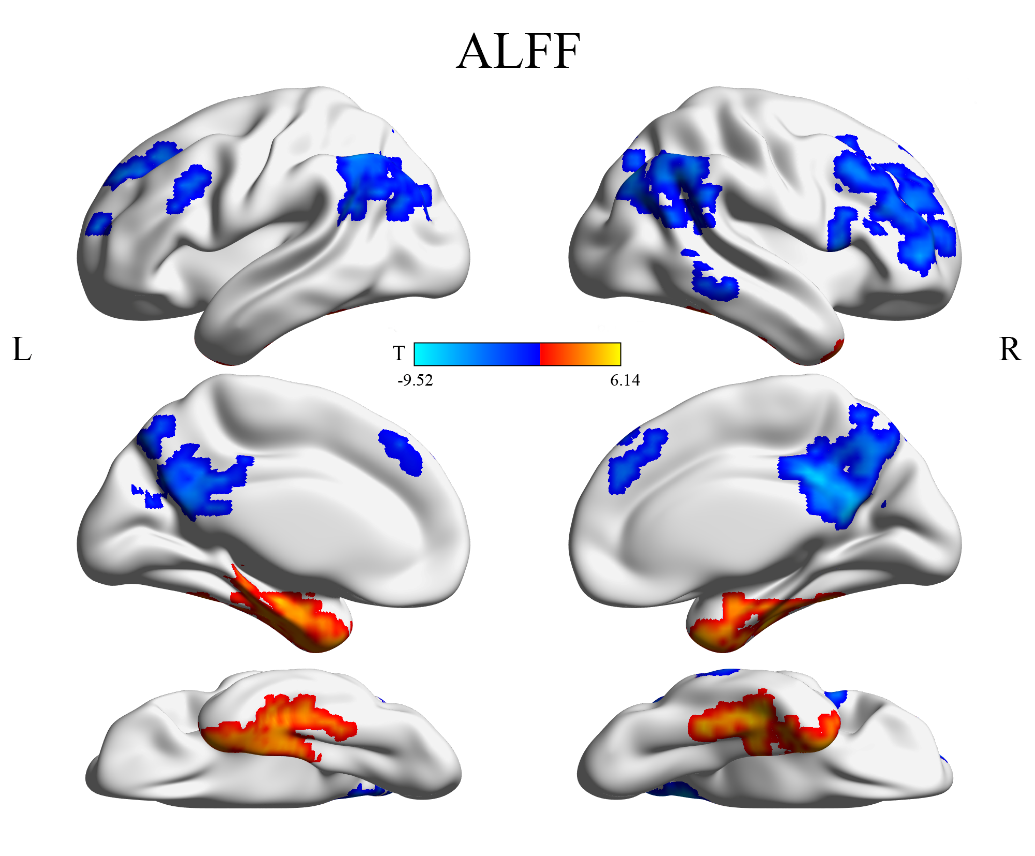


**Figure S2.** The ALFF differences between two groups (Smooth kernel was 4mm).


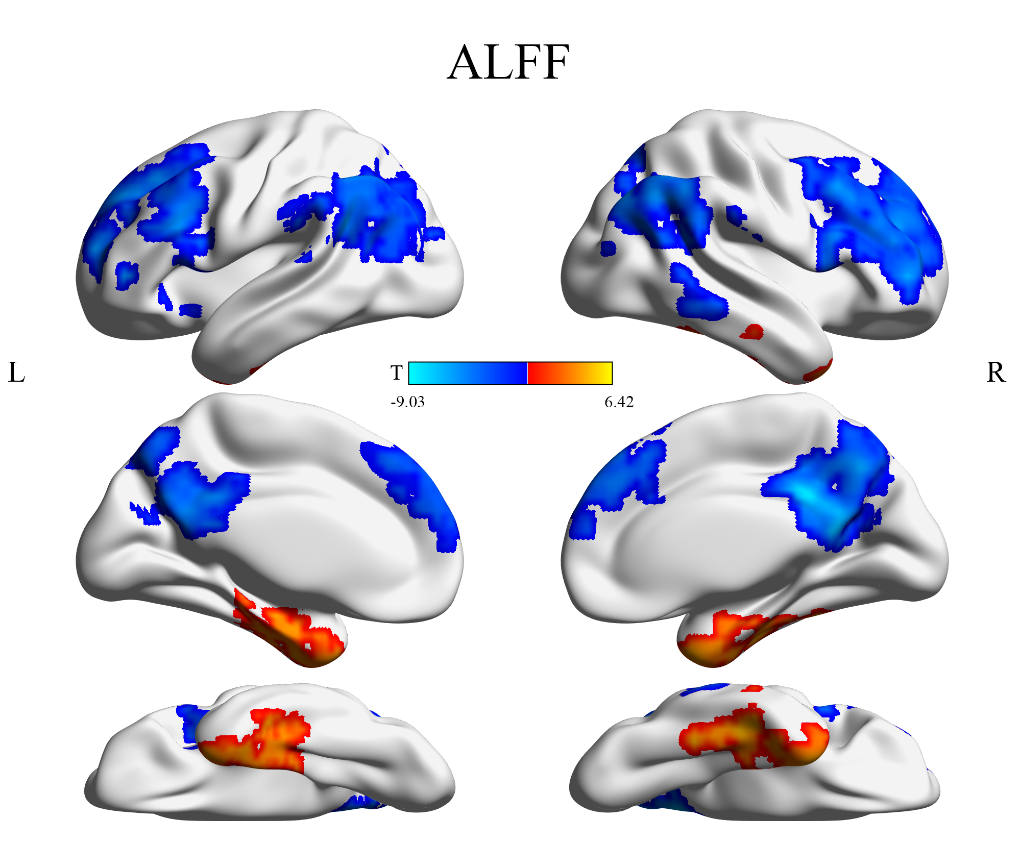


**Figure S3.** The ALFF differences between two groups (The global mean signals were regressed out in the preprocess).


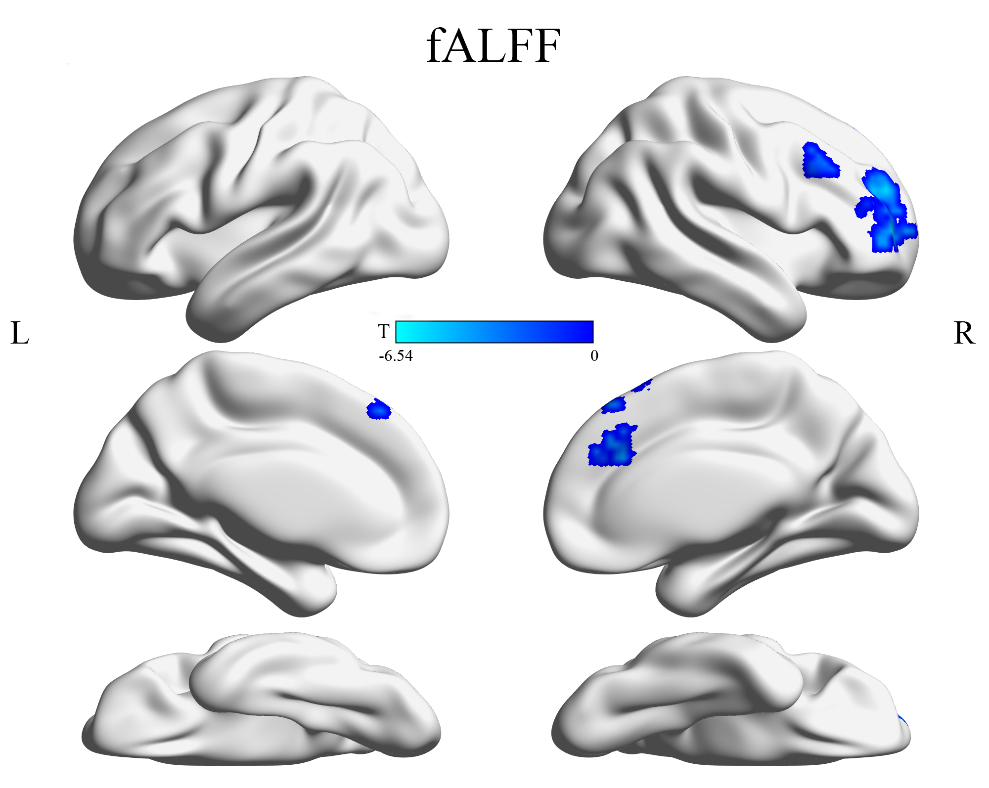


**Figure S4.** The fALFF differences between two groups (Frame-wise displacement (FD) parameters were regressed out in the statistical analysis).


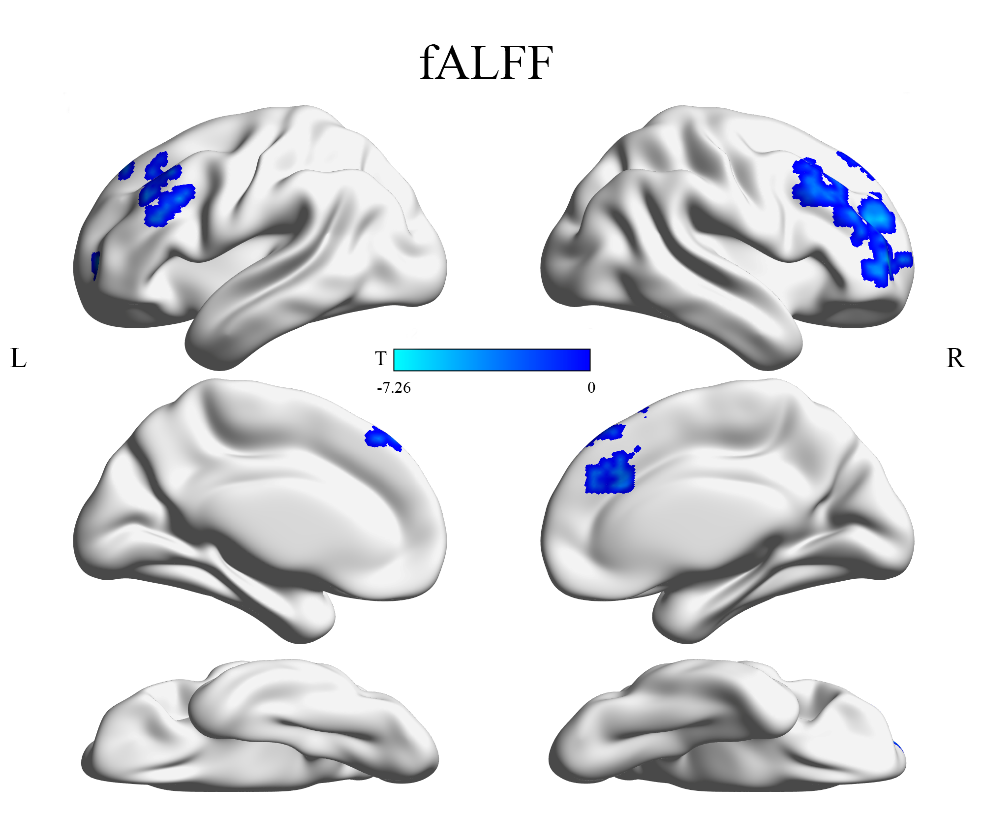


**Figure S5.** The fALFF differences between two groups (Smooth kernel was 4mm).


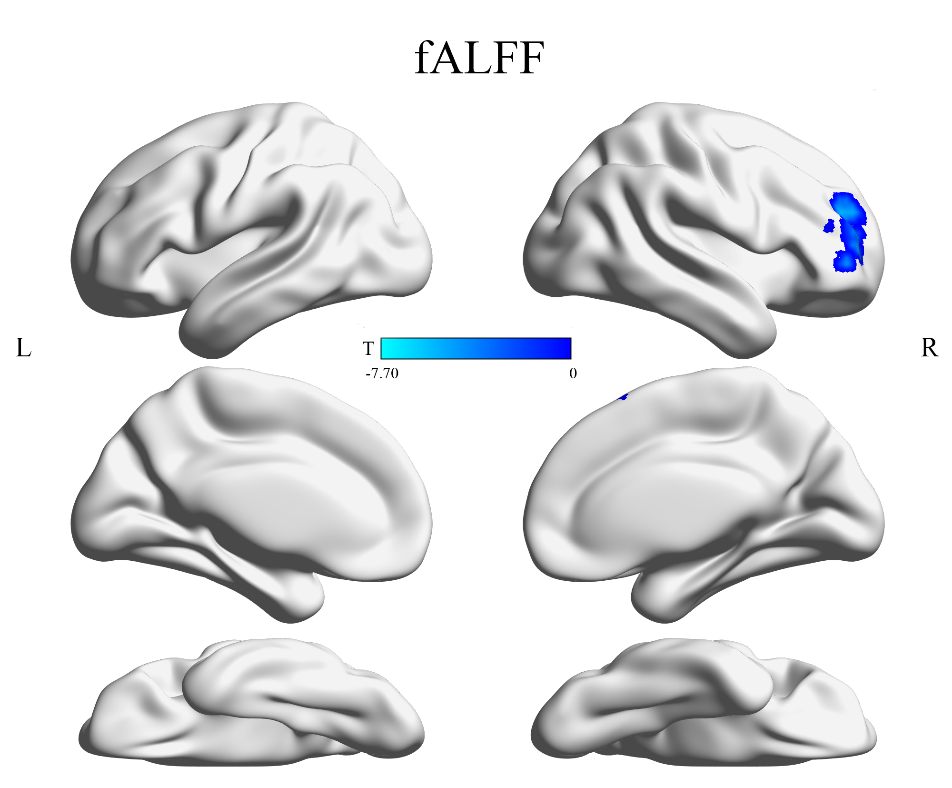


**Figure S6.** The fALFF differences between two groups (The global mean signals were regressed out in the preprocess).


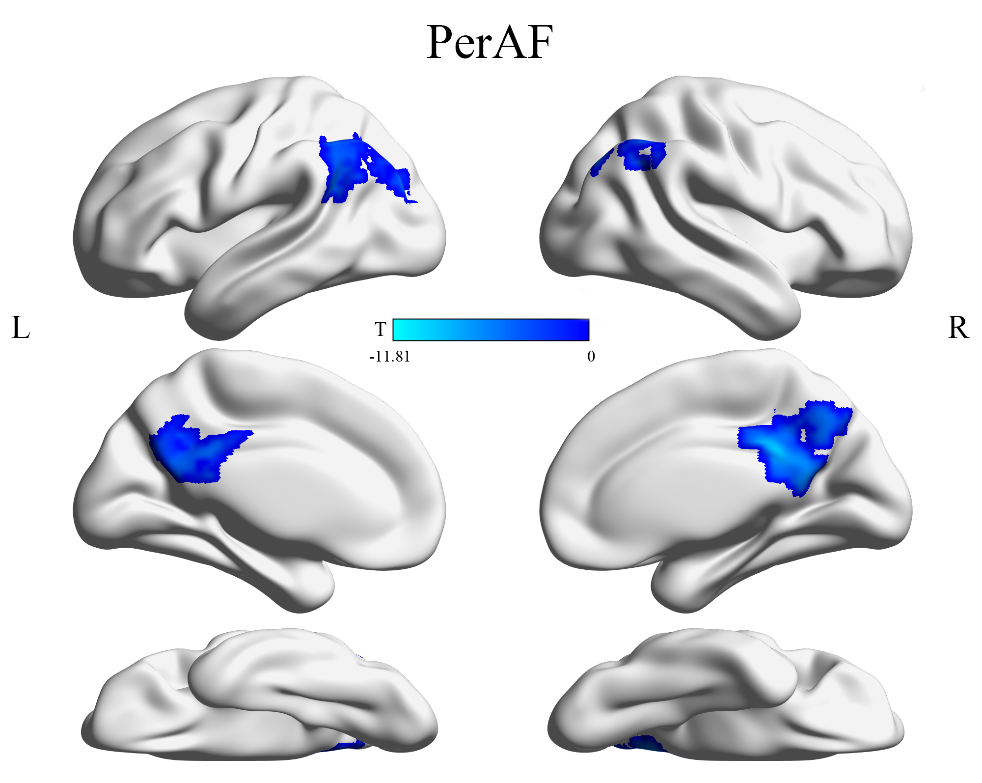


**Figure S7.** The PerAF differences between two groups (Frame-wise displacement (FD) parameters were regressed out in the statistical analysis).


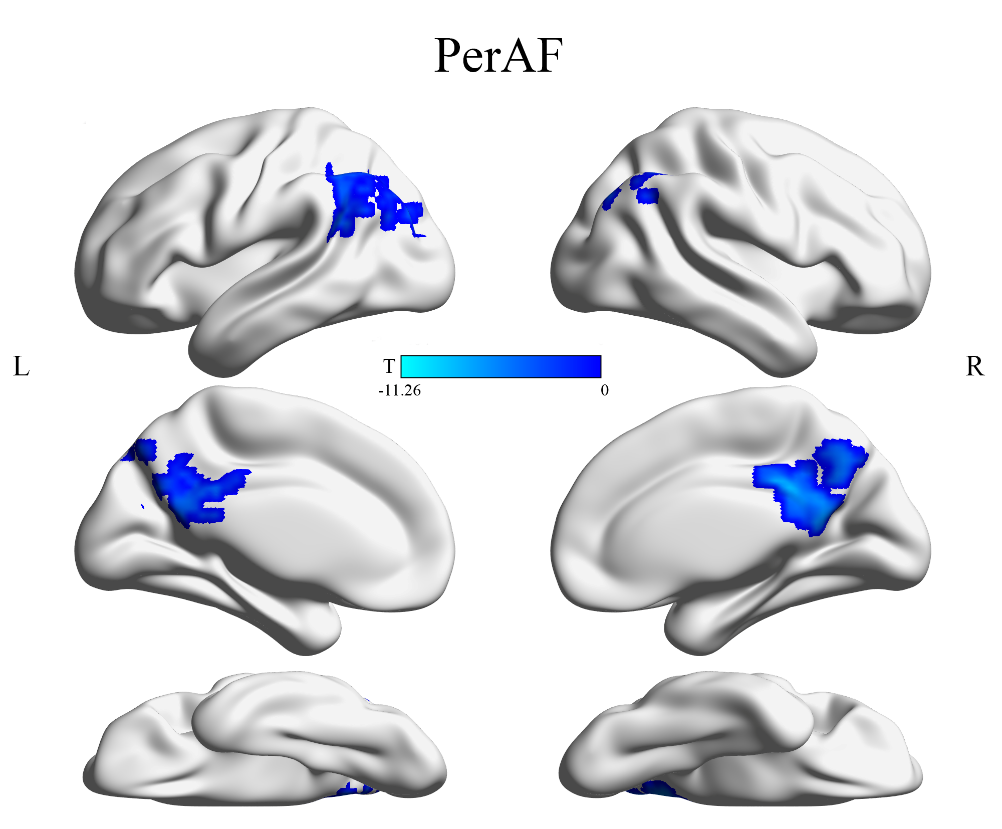


**Figure S8.** The PerAF differences between two groups (Smooth kernel was 4mm).


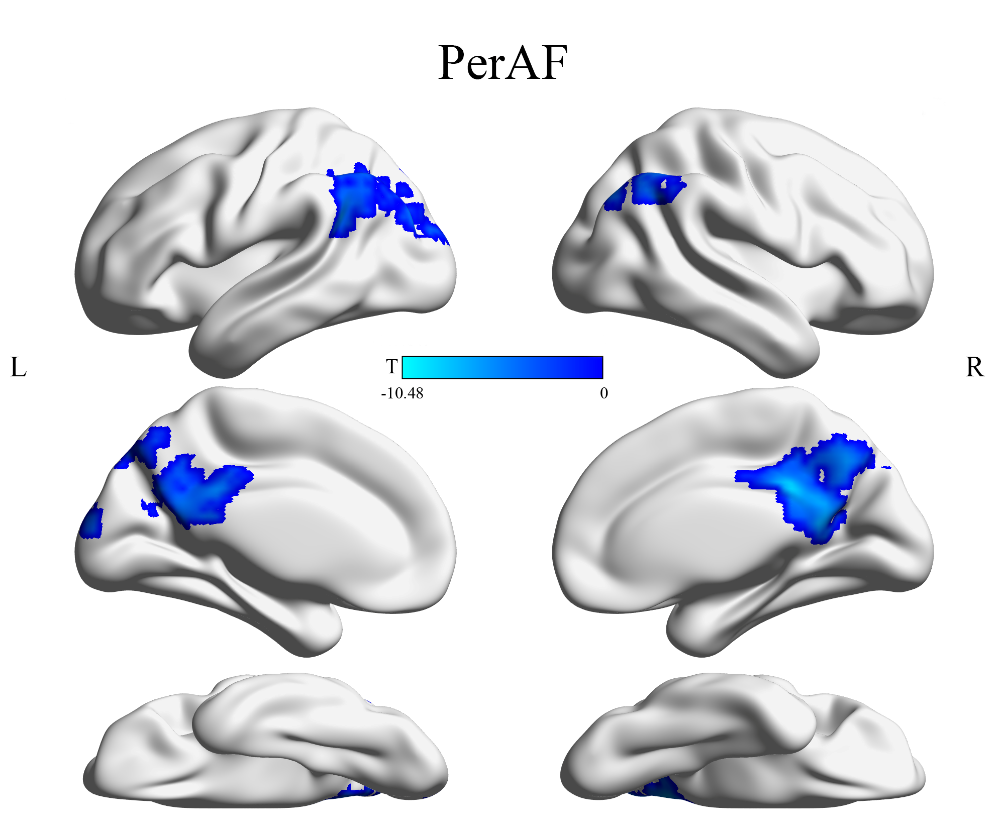


**Figure S9.** The PerAF differences between two groups (The global mean signals were regressed out in the preprocess)


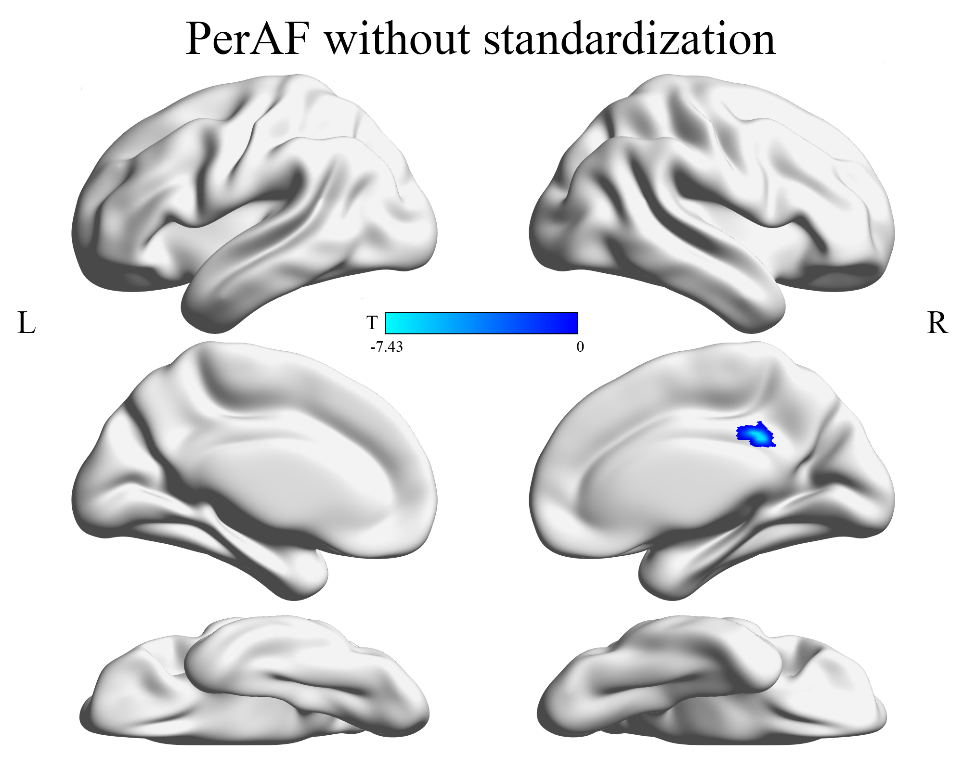


**Figure S10.** The PerAF (without standardization) differences between two groups (Frame-wise displacement (FD) parameters were regressed out in the statistical analysis).


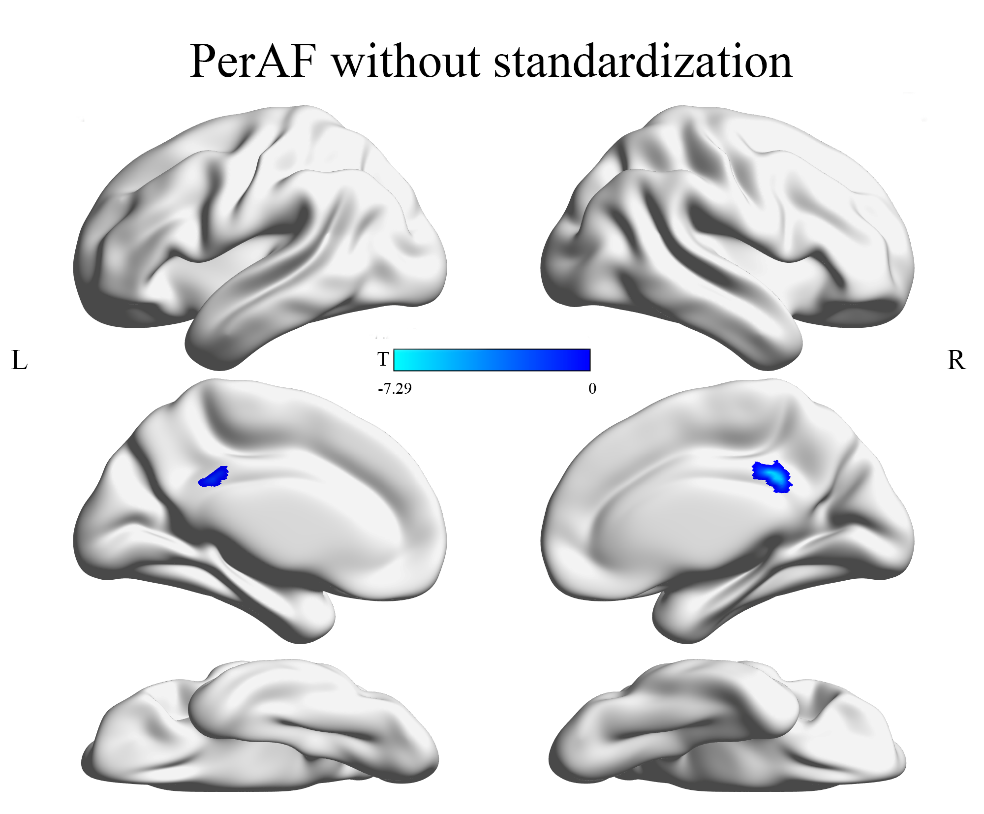


**Figure S11.** The PerAF (without standardization) differences between two groups (Smooth kernel was 4mm).


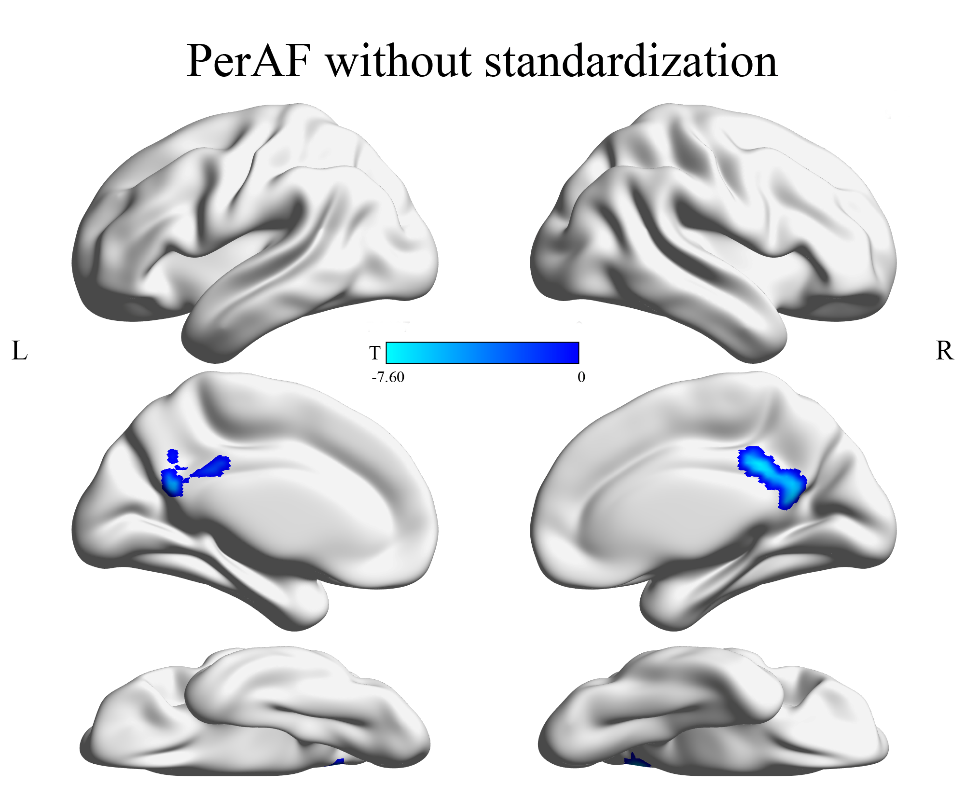


**Figure S12.** The PerAF (without standardization) differences between two groups (The global mean signals were regressed out in the preprocess).


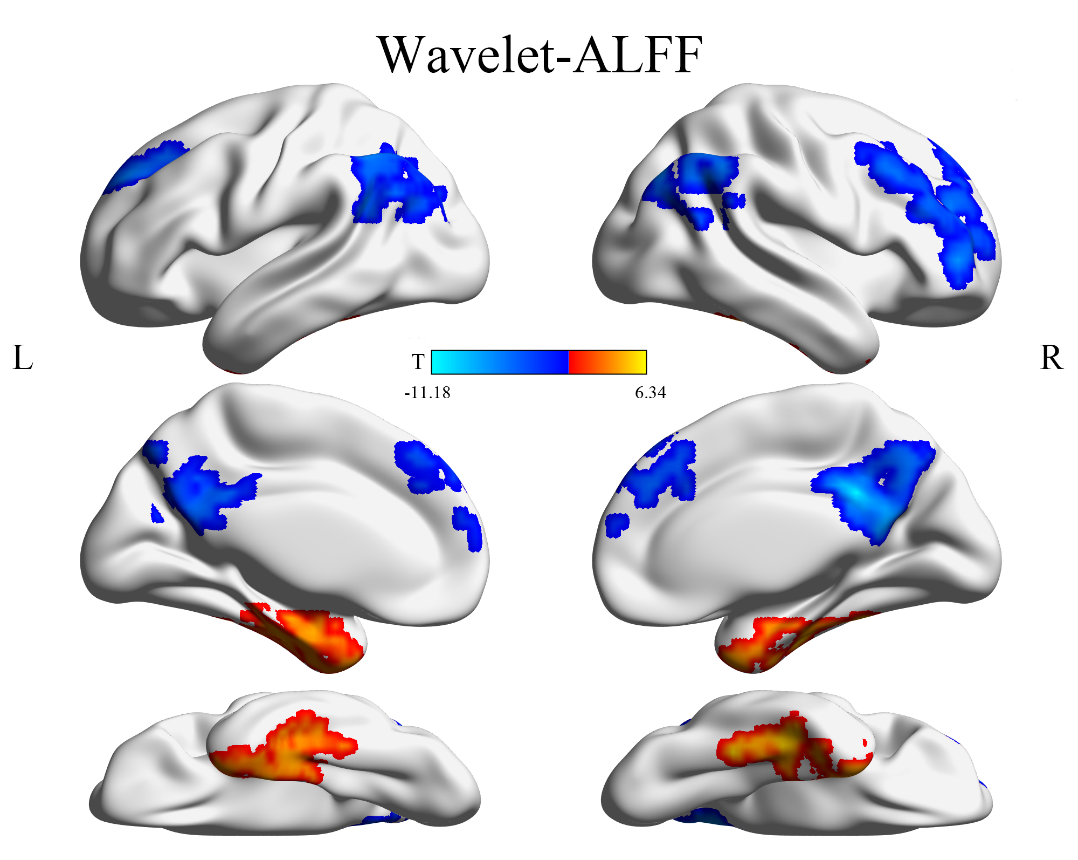


**Figure S13.** The Wavelet-ALFF differences between two groups (Frame-wise displacement (FD) parameters were regressed out in the statistical analysis).


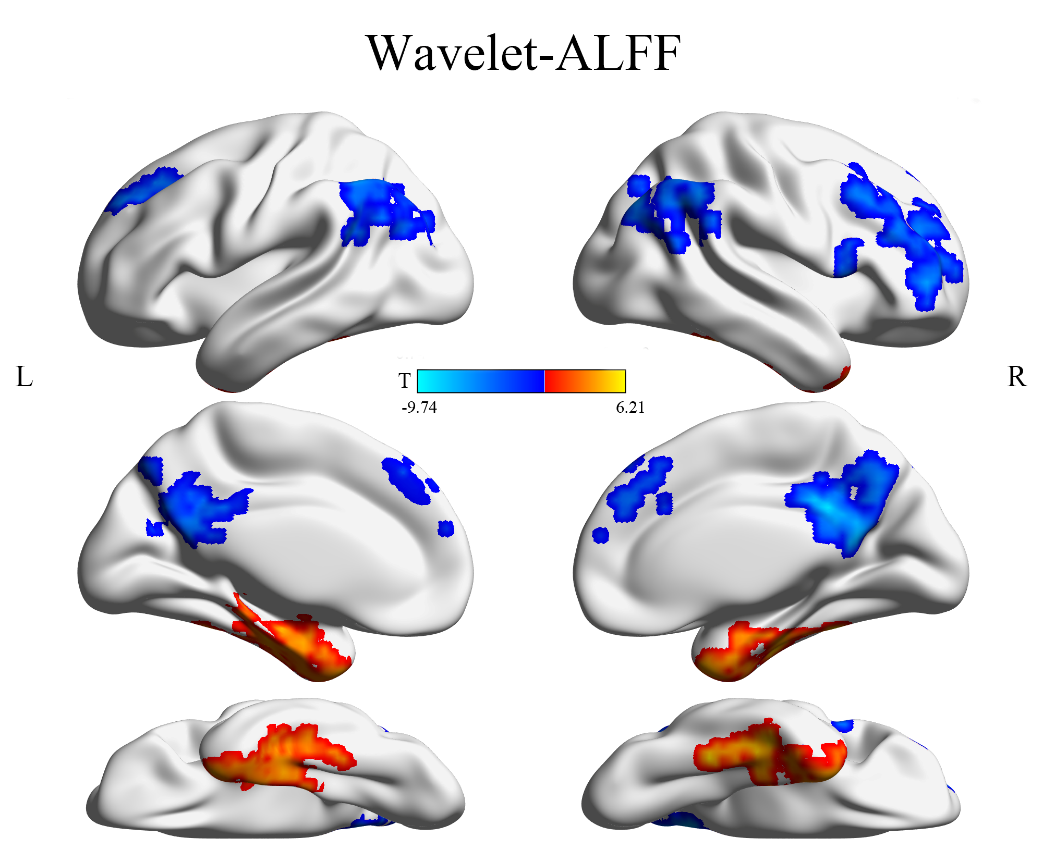


**Figure S14.** The Wavelet-ALFF differences between two groups (Smooth kernel was 4mm).


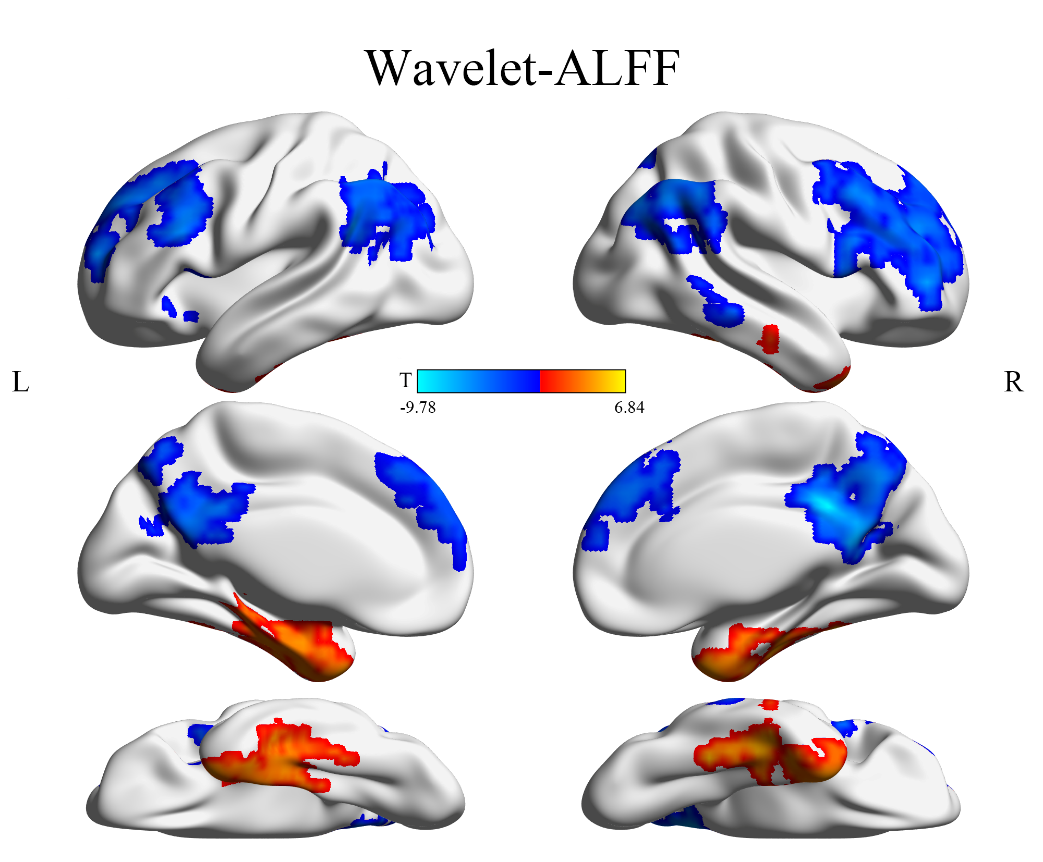


**Figure S15.** The Wavelet-ALFF differences between two groups (The global mean signals were regressed out in the preprocess).
